# A study of text-theoretical approach to S-box construction with image encryption applications

**DOI:** 10.1038/s41598-023-47607-6

**Published:** 2023-11-29

**Authors:** Abid Mahboob, Muhammad Nadeem, Muhammad Waheed Rasheed

**Affiliations:** Division of Science and Technology, Department of Mathematics, University of Education, Lahore, Pakistan

**Keywords:** Energy science and technology, Mathematics and computing

## Abstract

Data protection is regarded as one of the biggest issues facing companies that have been using public data for a long time. Numerous encryption techniques have been used to address these issues and safeguard data from any malicious attempts and assaults. A substitution box (S-box) is the basic component for modern block ciphers, which helps to ensure robust security of plain data while encryption and permits its lawful decipherment. The goal of this paper is to recommend an effective, original, and straightforward technique for the creation of robust S-boxes. A sample S-box is generated in the proposed work using the word “UNITY” but other words can also be used to generate many powerful S-boxes. The ASCII code is used to translate the word “UNITY” into binary form, after which a distinct matrix is constructed for each character of the word. In the next phase, a linear fractional transformation is constructed using these matrices, which is then utilized to generate the S-box. The constructed S-box was then evaluated against typical security standards to support its high cryptographic authority. The generated S-box's statistical and algebraic resilience is demonstrated by its very low linear probability and differential probability scores of 0.125 and 0.039, respectively, and a high non-linearity score of 111.5. To evaluate the effectiveness of the image encryption scheme, digital images are encoded using the created S-box. The performance and comparative research demonstrate that the suggested S-box is a real candidate for applications in the field of image encryption and has a stronger performance base.

## Introduction

Cryptography is a foundational technology that plays a pivotal role in safeguarding information and ensuring trust in digital interactions. Its importance extends across various domains, from securing online transactions to protecting national security and individual privacy. As the digital landscape continues to evolve, cryptography remains an essential tool for addressing the growing threats and challenges of the digital age. Many algorithms for protecting sensitive information are known in the literature. Ciphers used for plaintext encryption and cipher text decoding are classified into two types: block ciphers and stream ciphers^1,2^. The block cipher encrypts and decrypts data block by block. The data block is typically made up of one or more bytes. On the other hand, a stream cipher accomplishes these modifications by utilizing one bit or one byte in a single step. A block cipher has become the most potent approach for protecting sensitive data in contemporary cryptography^3^. Block ciphers are simpler to develop and more often used in practical security applications than stream ciphers^4^.

The substitution-permutation block ciphers are one of the most common types of block ciphers used to protect data. Permutation and substitution are important processes that are utilized in these ciphers to help change data into a mysterious format. The permutation function exchanges bits or bytes from the plaintext in the form of additional bits or bytes that are also included in the plaintext. In contrast, a nonlinear substitution process swaps out one block of data for another. S-box is used to replace the data in this instance^5,6^. A crucial component of modern block ciphers, an S-box considerably aids in the production of garbled output data (cipher text) from the provided input data (plaintext). To make things more difficult for the attackers, the S-box should provide a non-linear relationship between the input and output of the data^7^. If a substitution box may cause greater uncertainty for the attackers, the block cipher is regarded as more secure. On the other hand, a weak S-box can undermine the security of a block cipher. A weak S-box has low non-linearity, meaning it does not effectively obscure the relationship between input and output bits. In other words, it doesn’t introduce sufficient complexity in the substitution operation, making it vulnerable to various cryptanalysis techniques. The avalanche effect is a desirable property in cryptography where a small change in the input should result in a significantly different output. Weak S-boxes often fail to exhibit a strong avalanche effect, meaning small input changes may lead to small or predictable output changes, which can be exploited by attackers. Weak S-boxes may exhibit linear or affine structures, making it easier for cryptanalysts to derive mathematical relationships between the input and output. This can lead to efficient attacks, such as linear and differential cryptanalysis. The security provided by a block cipher using the substitution box depends entirely upon the effectiveness of the corresponding S-box^8,9^.

As an outcome, researchers eventually developed novel methods for building key-dependent, dynamic S-boxes. Among these algebraic ideas, linear fractional transformation (LFT) is an essential method for constructing robust S-boxes. The LFT approach was employed by the authors in^10–13^ to construct efficient and reliable S-boxes. When measured against the accepted cryptographic standards that are used to assess an S-box, the created S-boxes showed respectable results. In addition to LFT approaches, researchers have also suggested effective methods for producing S-boxes. For example, authors in^14^ offered a method that made use of the concept of cubic fractional transformation (CFT) to create effective S-boxes. Another straightforward method has been proposed in^15^ for constructing S-boxes by using cubic polynomial transformation. For constructing strong S-boxes, the use of this approach is incredibly straightforward, effective, and efficient. In^16^, a novel method for building robust S-boxes with linear transformation was put forward. Using the permutation process, this method strengthens the resulting S-box. DNA computing is also employed by authors to construct resilient S-boxes and cryptographically robust ciphers using the techniques provided in^17–22^. These S-boxes and ciphers were shown to be strong in terms of cryptographic standards and resistance to various assaults through analysis. Due to the unpredictability of chaotic systems, chaos is also an important field exploited in cryptography for creating reliable S-boxes^20^. Chaos theory was utilized by several scholars^23–26^ to construct S-boxes with strong cryptographic properties. Hyperchaotic schemes were employed by the authors of^27,28^ to build robust and reliable S-boxes. Many other researchers have also developed S-boxes using a variety of different methods, including cellular automata^29^, graph theory^30^, elliptic curve^31^, and optimization approaches^32^, etc.

The construction of S-boxes relies on certain key assumptions to ensure their security and effectiveness. Here are some key assumptions for the construction of S-boxes:

- S-boxes should exhibit strong non-linearity, which means that they should not be easily expressible as a linear function of their inputs. This property helps prevent attackers from exploiting algebraic relationships between input and output bits.
- S-boxes should satisfy the avalanche effect, where a change in a single input bit should cause approximately half of the output bits to change on average. This property ensures that small changes in the plaintext result in substantial changes in the cipher text.
- S-boxes should be designed to resist known cryptanalysis techniques such as differential and linear cryptanalysis. These techniques attempt to find patterns or correlations between plaintext, cipher text, and key bits, and strong S-boxes can help thwart these attacks.
- S-boxes should be bijective, meaning that each input value maps to a unique output value, and vice versa. This property ensures that the S-box can be inverted, allowing for decryption.

In this manuscript, a text-theoretical approach is used for the construction of effective S-boxes. Text encryption by using the Adjacency matrix of Graphs is used in different branches of the research and is frequently utilized to address a wide range of practical issues. Mathematicians are becoming more and more aware of the importance of graph theory. It is employed in electrical engineering, solid-state physics, organic chemistry, statistical mechanics, operations research, and optimization theory. Numerous other fields, including computer science, mathematics, and engineering, are impacted by the study of cryptography^33–37^.

The significant contributions provided in this study are as follows:

- To generate the initial S-boxes, a novel and straightforward Graph-theoretic method is proposed, and subsequently its nonlinearity is enhanced by employing a permutation of symmetric group.
- Conventional S-box assessment criteria and the S-boxes already accessible in the existing literature are used for analyzing the proposed S-box. The significant contribution of the planned S-box is confirmed by this performance investigation.
- The suggested method's sample S-box is used for image encryption. The S-box-based encryption has outstanding encryption effectiveness, according to simulation and effectiveness evaluations.

The subsequent sections of the paper are structured as follows. The construction of S-boxes utilizing graphical techniques is thoroughly covered in section “Algebraic structure of S-box”. Section “Algebraic analysis” compares the performance of S-box created using the suggested approach to currently in use modern S-boxes. In section “Image encryption”, the suggested S-box is tested and evaluated for use in image encryption. In contrast, section “Conclusion” brings the study's conclusion.

## Algebraic structure of S-box

**Figure 1:**
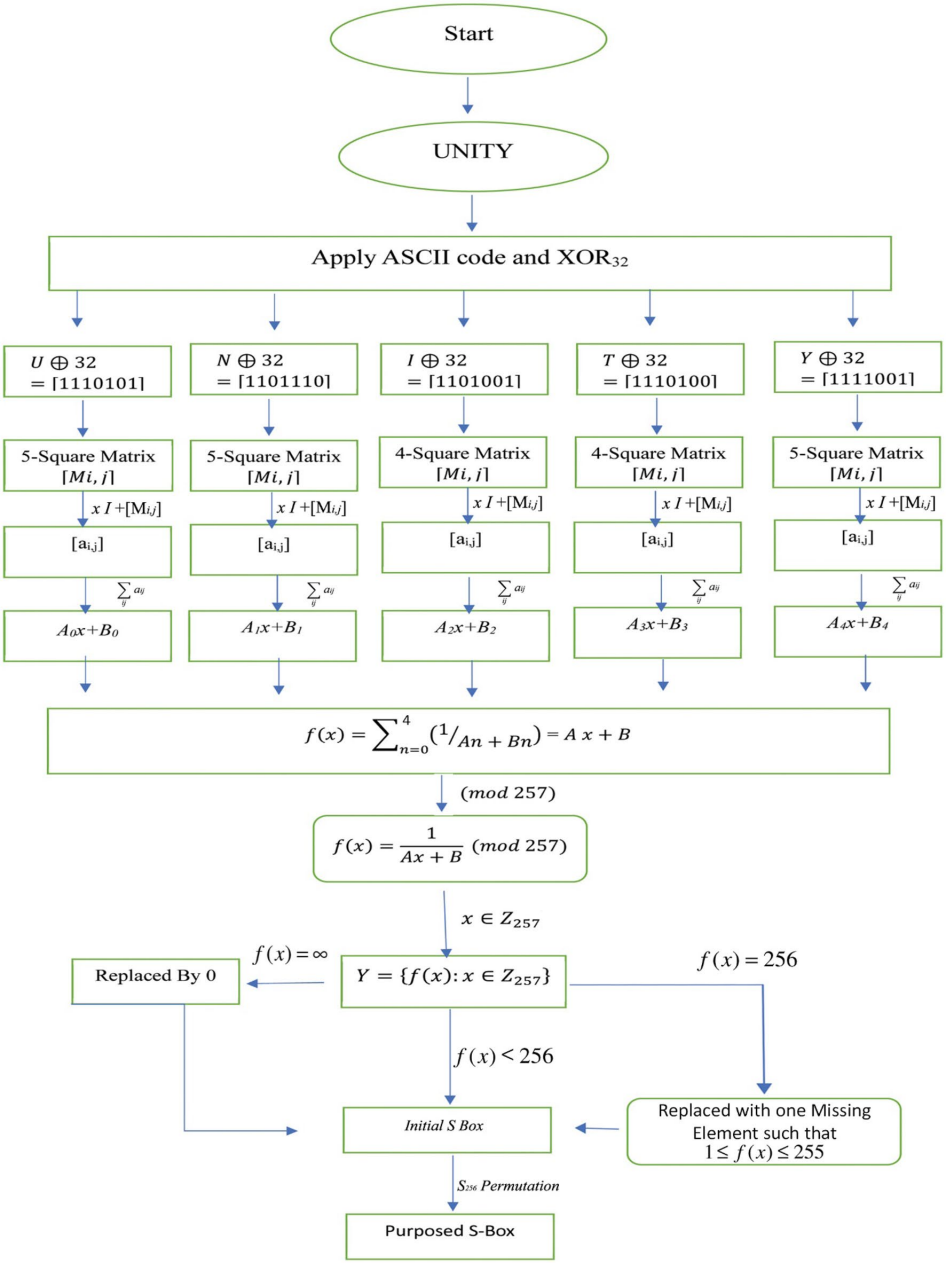
A flow chart for the construction of S-box.

For the purpose of constructing substitution boxes, the suggested method essentially consists of three stages. First, LFT is generated from a phrase using the concepts of graph theory. Second, this LFT generates an S-box, and finally, a symmetric group permutation is used to enhance the non-linearity of the initially generated S-box. The suggested algorithm’s initial phase is based on graph theory because text characters are hidden in the graph according to the graph's vertices and their degrees. Every character in a text is first changed to its ASCII code, and then it is turned to binary notation. The order of the matrix is then determined by evaluating XOR for each character of the given text, which determines the order of the matrix for each character individually. The number of rows and columns in a matrix corresponds to the number of nodes in a graph, and we follow the same procedure to create a graph using a matrix as the definition of the adjacency matrix of a graph. Each step of the proposed approach to constructing S-boxes is given in algorithm 2.1 and the flow chart for the construction of the S-box is given in Fig. 1.

### Algorithm 2.1:

1. Input can be any word or phrase.
2. Convert each character of the message into its ASCII code in binary format.
3. Compute XOR_32_ for each binary number.
4. For each binary number, form a one-dimensional array by counting the number of 1’s.
5. Construct a square matrix $\left[{M}_{i,j}\right]$ with an order equal to the number of 1's in the array.
6. The diagonal elements of $\left[{M}_{i,j}\right]$ will be equal to zero.
7. For every binary number, count the number of 0’s following 1’s.
8. If the binary stream ends with 1 and is followed by 0, then put ${m}_{i,j}={m}_{j,i}=n+1$, where n is the number of 0’s following 1.
9. All elements are stored in the upper diagonal of the matrix, and the matrix that is forming is a real symmetric matrix.
10. On finding the last element of binary stream 1, put 1 in the first entry of the last column of a matrix.
11. The above process is repeated until we are done with the binary stream for each character.
12. Create a square matrix $xI$ for all matrices $\left[{M}_{i,j}\right]$ with the same order as that of the matrix $\left[{M}_{i,j}\right]$ where $I$ is the identity matrix and $x$ is its vector multiple.
13. Determine the sum of each matrix combination as, $xI+\left[{M}_{i,j}\right]=[{a}_{i,j}]$.
14. Determine the grand sum $\sum_{i,j}{a}_{i,j}$ of these matrices that will be linear functions.
15. Find the sum of all these linear functions and take its reciprocal to get an LFT of the type $\frac{1}{Ax+B}$.
16. Utilize this LFT to construct the initial S-box.
17. Apply the appropriate symmetric group permutation to increase the nonlinearity of this initially created S-box.
18. Output is a suggested S-box with robust algebraic characteristics.

### Construction of a specimen S-box

Let's say that we wanted to create an S-box from the word “UNITY.” Using the ASCII table, we were able to find the binary format of all of its alphabets, as shown in Table 1.

**Table 1:** Binary format for each character of the word “UNITY”.

Alphabet	U	N	I	T	Y
ASCII Code	85	78	73	84	89
Binary number	1010101	1001110	1001001	1010100	1011001
XOR	1110101	1101110	1101001	1110100	1111001

Use the algorithm 2.1 to create a square symmetric matrix for each letter, as shown in Table 2.

**Table 2:** LFT computation table.

Alphabets	Adjacency Matrices $\left[{M}_{i,j}\right]$	Graph	$xI+\left[{M}_{i,j}\right]=[{a}_{i,j}]$	$\sum_{i,j}{a}_{i,j}$
U	$$\left[\begin{array}{cc}\begin{array}{cc}0& 1\\ 1& 0\\ 0& 1\end{array}& \begin{array}{ccc}0& 0& 1\\ 1& 0& 0\\ 0& 2& 0\end{array}\\ \begin{array}{cc}0& 0\\ 1& 0\end{array}& \begin{array}{ccc}2& 0& 2\\ 0& 2& 0\end{array}\end{array}\right]$$		$$\left[\begin{array}{cc}\begin{array}{cc}x& 1\\ 1& x\\ 0& 1\end{array}& \begin{array}{ccc}0& 0& 1\\ 1& 0& 0\\ x& 2& 0\end{array}\\ \begin{array}{cc}0& 0\\ 1& 0\end{array}& \begin{array}{ccc}2& x& 2\\ 0& 2& x\end{array}\end{array}\right]$$	$5x+14$
N	$$\left[\begin{array}{cc}\begin{array}{cc}0& 1\\ 1& 0\\ 0& 2\end{array}& \begin{array}{ccc}0& 0& 2\\ 2& 0& 0\\ 0& 1& 0\end{array}\\ \begin{array}{cc}0& 0\\ 2& 0\end{array}& \begin{array}{ccc}1& 0& 1\\ 0& 1& 0\end{array}\end{array}\right]$$		$$\left[\begin{array}{cc}\begin{array}{cc}x& 1\\ 1& x\\ 0& 2\end{array}& \begin{array}{ccc}0& 0& 2\\ 2& 0& 0\\ x& 1& 0\end{array}\\ \begin{array}{cc}0& 0\\ 2& 0\end{array}& \begin{array}{ccc}1& x& 1\\ 0& 1& x\end{array}\end{array}\right]$$	$5x+14$
I	$$\left[\begin{array}{cc}\begin{array}{cc}0& 1\\ 1& 0\end{array}& \begin{array}{cc}0& 1\\ 2& 0\end{array}\\ \begin{array}{cc}0& 2\\ 1& 0\end{array}& \begin{array}{cc}0& 3\\ 3& 0\end{array}\end{array}\right]$$		$$\left[\begin{array}{cc}\begin{array}{cc}x& 1\\ 1& x\end{array}& \begin{array}{cc}0& 1\\ 2& 0\end{array}\\ \begin{array}{cc}0& 2\\ 1& 0\end{array}& \begin{array}{cc}x& 3\\ 3& x\end{array}\end{array}\right]$$	$4x+14$
T	$$\left[\begin{array}{cc}\begin{array}{cc}0& 1\\ 1& 0\end{array}& \begin{array}{cc}0& 3\\ 1& 0\end{array}\\ \begin{array}{cc}0& 1\\ 3& 0\end{array}& \begin{array}{cc}0& 2\\ 2& 0\end{array}\end{array}\right]$$		$$\left[\begin{array}{cc}\begin{array}{cc}x& 1\\ 1& x\end{array}& \begin{array}{cc}0& 3\\ 1& 0\end{array}\\ \begin{array}{cc}0& 1\\ 3& 0\end{array}& \begin{array}{cc}x& 2\\ 2& x\end{array}\end{array}\right]$$	$4x+14$
Y	$$\left[\begin{array}{cc}\begin{array}{cc}0& 1\\ 1& 0\\ 0& 1\end{array}& \begin{array}{ccc}0& 0& 1\\ 1& 0& 0\\ 0& 1& 0\end{array}\\ \begin{array}{cc}0& 0\\ 1& 0\end{array}& \begin{array}{ccc}1& 0& 3\\ 0& 3& 0\end{array}\end{array}\right]$$		$$\left[\begin{array}{cc}\begin{array}{cc}x& 1\\ 1& x\\ 0& 1\end{array}& \begin{array}{ccc}0& 0& 1\\ 1& 0& 0\\ x& 1& 0\end{array}\\ \begin{array}{cc}0& 0\\ 1& 0\end{array}& \begin{array}{ccc}1& x& 3\\ 0& 3& x\end{array}\end{array}\right]$$	$5x+14$

We now find the desired LFT as shown below by taking the reciprocal of the sum of all the mappings in the last column.

1 $$ f\left( x \right) = \frac{1}{{\left( {5x + 14} \right) + \left( {5x + 14} \right) + \left( {4x + 14} \right) + \left( {4x + 14} \right) + \left( {5x + 14} \right)}} $$

2 $$ f\left( x \right) = \frac{1}{23x + 70} $$

Now enter the values from 0 to 255 into the mapping provided in Eq. (2) and to keep all the outputs in the range 0 to 255 replace $f(64)$ with 0 and $f(131)$ with 191. After resolving all of the outputs under mod 257, we find the initial S-box as shown in Table 3.

**Table 3:** Initially created S-box from Eq. (2).

246	152	113	98	211	232	236	168	171	90	6	74	26	218	99	122
71	160	197	110	241	145	228	127	188	206	252	106	9	68	70	150
235	226	165	215	85	12	52	198	142	137	225	199	119	247	18	140
213	73	170	104	27	193	238	36	169	83	54	219	81	108	162	67
0	190	95	149	176	38	203	174	88	221	19	64	230	153	87	184
44	117	239	10	138	58	32	120	115	59	205	245	172	42	92	31
22	107	187	189	248	151	5	51	69	130	29	112	16	147	60	97
186	135	158	231	183	251	167	86	89	21	25	46	159	144	105	11
82	182	39	222	40	223	209	124	242	204	191	131	156	154	100	163
253	65	250	143	229	56	14	8	57	202	132	30	96	177	2	93
47	196	207	79	180	148	179	244	63	220	33	254	155	212	136	43
195	173	24	139	17	141	237	23	146	208	129	72	166	181	216	134
123	41	76	91	185	128	49	111	234	20	116	240	118	233	84	62
214	121	45	102	3	224	37	194	13	78	109	77	178	50	61	210
164	255	80	161	227	125	55	200	249	243	201	28	114	7	192	4
94	157	103	101	126	66	53	15	133	48	34	217	35	1	75	175

This S-box’s Boolean functions have an average non-linearity value of 99.75, though a resilient S-box could be created by using the Symmetric group permutation to increase this value. The 256 cells of Table 3 are subjected to the following symmetric group permutation in this instance. As a result, we find in Table 4 a sturdy S-box with an average non-linearity of 111.5.

**Table 4:** Proposed S-box.

5	248	146	244	85	251	110	225	187	138	254	84	30	208	109	125
130	46	54	64	238	186	234	103	29	49	74	45	7	188	68	79
193	212	95	222	194	209	76	176	111	61	156	215	112	159	62	48
128	23	38	55	195	150	213	116	224	184	19	50	20	12	148	131
1	240	182	202	33	100	80	121	77	214	206	189	171	169	134	221
203	26	11	86	155	114	137	247	241	123	31	154	0	42	65	83
2	219	218	39	249	151	250	235	129	126	236	59	16	135	205	97
37	227	34	216	117	157	18	140	67	71	149	13	168	73	4	22
63	53	105	28	106	166	255	118	115	204	226	179	211	43	185	165
231	99	120	158	9	102	239	133	3	15	75	245	58	101	72	57
232	66	40	10	132	41	246	180	230	210	78	196	220	88	198	181
162	229	98	94	81	199	174	124	217	25	153	122	191	167	127	177
104	32	197	142	119	252	253	160	56	21	96	173	92	200	145	44
136	228	89	90	143	201	163	242	70	172	93	233	183	113	192	35
69	82	223	139	27	36	47	141	91	175	108	14	144	52	60	237
207	24	170	243	51	147	8	164	17	190	107	152	6	161	87	178

**Permutation of **$${{\varvec{S}}}_{256}$$**:** (1 167 140 64 121 211 28 133 163 241 180 228 254 65 93 218 171 69 40 175 209 74 80 58 96 91 111 239 223 42 87 194 166 63 177 53 229 114 110 246 162 172 11 253 224 170 173 85 10 212 150 201 23 210 72 183 240 127 131 100 76 20 7 107 25 30 31 217 124 18 200 41 196 233 101 2 252 185 3 222 60 98 251 115 148 213 153 160 219 15 146 95 205 136 184 50 126 237 86 157 203 56 230 16 188 159 97 128 83 151 236 132 36 44 182 232 206 220 73 174 34 139 189 134 227 71 81 208 47 119 190 176 142 92 156 13 82 117 221 256 234 244 158 192 79 255 155 165 168 4 179 242 118 6 161 231 52 193 90 108 45 197 143 70 51 243 24 191 116 145 199 26 75 59 19 195 39 238 29 149 178 204 66 250 48 120 84 164 32 54 33 104 245 106 17 122 202 61 181 249 152 247 130 67 35 144 215 113 22 207 12 27 198 49 55 21 89 137 216 37 5 141 43 8 125 46 88 147 103) (9 77 169 129 226 135 38 62 235 214 57 78 187 105 225 248 154 68 123 186 14 99) (94) (102) (109) (138) (112).

## Algebraic analysis

For effective encryption, an S-box must have robust algebraic properties that enable nonlinear associations between the input and output of data. This section carefully analyzes the created S-box given in Table 4 using the following standards for judging the cryptographic potency of substitution boxes.

- Bijective
- Nonlinearity
- Bit Independence Criterion
- Strict Avalanche Criterion
- Linear Probability
- Differential Uniformity

As a comparison tool between our suggested S-box and several current S-boxes, we additionally choose recently explored S-box approaches.

### Bijective

If a mapping has a unique output against a particular input, it is said to be bijective. This characteristic should be very clearly demonstrated by an S-box design. In the instance of 8 × 8 S-box, every value entered in the interval [0, 255] should result in a unique value for output in the interval [0, 255]. This condition is validated by the created S-box in Table 4 since it has all conceivable different output values in the interval [0, 255]. Additionally, the number of 1’s in each Boolean function is the same as the number of 0’s.

### Non-linearity

An S-box design is thought to be better if it possesses the capacity to convert the given inputs into outputs nonlinearly. This kind of S-box is useful for thwarting intruders’ attempts at linear cryptanalysis. Using Eq. (3) as provided by^38^, one may determine the nonlinearity of Boolean functions:

3 $$ NL_{f} = 2^{n - 1} \left( {1 - 2} \right.^{ - n} max\left| {W_{f} \left( a \right)} \right| $$

**Figure 2:**
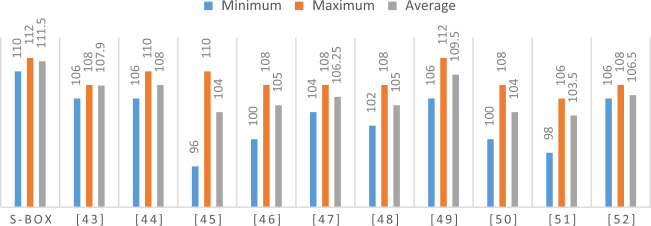
A comparison between NL scores of different S-boxes.

Here $W_{f} \left( a \right) = \mathop \sum \limits_{{a \in F_{2}^{n} }} \left( { - 1} \right)^{f\left( x \right) \oplus a.x)}$ is the value of Walsh Spectrum. Eight balanced Boolean functions that make up our predicted S-box have non-linearity values of 112, 112, 112, 110, 112, 112, 112, and 110. It is clear that the lowest, highest, and mean nonlinearity values for our S-box are, respectively, 110, 112, and 111.75. Figure 2 shows a graphical comparison of our S-box's nonlinearity results with those of newly developed S-box construction methods. The contrast shows that our predicted S-box's nonlinearity value works better than the nonlinearity outcomes of numerous other different existing S-boxes.

### Strict Avalanche Criterion (SAC)

This S-box feature guarantees that one input bit shift modifies 50% of the output bits^39^. As a result, a powerful S-box has a SAC score that is roughly equivalent to 0.5. Table 5 provides the dependence matrix of our S-box’s SAC readings. The mean SAC score of our S-box is 0.4978, which is very close to 0.5. As a result, our anticipated S-box adequately fulfills the SAC requirement. Table 8's comparison of the predicted S-box’s SAC values with those of other S-boxes shows that our S-box’s SAC score is gracefully consistent with those of other S-boxes.

**Table 5:** Strict Avalanche values of created S-box.

0.5	0.5	0.4688	0.4688	0.5156	0.5156	0.4844	0.5
0.5	0.5312	0.5469	0.4844	0.5	0.5469	0.4531	0.5
0.5312	0.5	0.4531	0.5156	0.4844	0.4844	0.5625	0.4531
0.5	0.5312	0.4688	0.4844	0.5156	0.5156	0.5	0.4844
0.4844	0.5156	0.5	0.4688	0.4844	0.4844	0.4844	0.5156
0.4688	0.5	0.4844	0.5312	0.4844	0.4688	0.4688	0.4688
0.5	0.5312	0.5	0.4531	0.5	0.4688	0.4531	0.5469
0.5156	0.4844	0.4844	0.4844	0.5781	0.5	0.5	0.5469

### Bit Independence Criterion (BIC)

This feature of an S-box guarantees that when just a single input bit is changed, alterations in any two output bits do not rely on one another^39^. The BIC-SAC and BIC nonlinearity (BIC-NL) values of the created S-box are shown in Tables 6 and 7, respectively.

**Table 6:** BIC-SAC values.

0	0.4805	0.4824	0.4941	0.4863	0.5156	0.4863	0.498
0.4805	0	0.4785	0.4844	0.4941	0.5	0.5195	0.4648
0.4824	0.4785	0	0.4863	0.4961	0.5215	0.4961	0.5215
0.4941	0.4844	0.4863	0	0.4922	0.498	0.4785	0.498
0.4863	0.4941	0.4961	0.4922	0	0.4961	0.5234	0.4805
0.5156	0.5	0.5215	0.498	0.4961	0	0.5039	0.5078
0.4863	0.5195	0.4961	0.4785	0.5234	0.5039	0	0.5137
0.498	0.4648	0.5215	0.498	0.4805	0.5078	0.5137	0

**Table 7:** BIC nonlinearity values.

0	104	108	108	106	96	104	104
104	0	106	106	100	106	104	108
108	106	0	104	104	100	106	100
108	106	104	0	106	100	106	104
106	100	104	106	0	104	104	100
96	106	100	100	104	0	102	102
104	104	106	106	104	102	0	106
104	108	100	104	100	102	106	0

BIC-NL and BIC-SAC average values for the constructed S-box are 103.86 and 0.4964, respectively. These results suggest that the predicted S-box fully satisfies the BIC criterion. Moreover, Table 8 provides an investigation of BIC values for various S-boxes.

**Table 8:** A comparison between algebraic properties of different well-known S-boxes.

S-box Method	BIC-NL	SAC	LP	DP
Proposed	103.86	0.4978	0.125	0.039
^42^	103.1	0.509	0.1406	0.039
^43^	102.9	0.499	0.141	0.047
^44^	103.0	0.493	0.125	0.031
^45^	103.0	0.500	0.125	0.047
^46^	103.6	0.501	0.139	0.039
^47^	102.9	0.503	0.1484	0.047
^48^	106.9	0.507	0.1328	0.031
^49^	102.6	0.497	0.137	0.039
^50^	103.5	0.496	0.1328	0.055
^51^	104.1	0.501	0.1328	0.039
^52^	106.1	0.509	0.113	0.031
^53^	103.9	0.503	0.1328	0.039
^54^	103.6	0.499	0.125	0.039
^55^	103.9	0.500	0.109	0.039
^56^	103.5	0.506	0.125	0.039
^57^	102.3	0.493	0.141	0.047

### Linear probability (LP)

The bits of plaintext must be mixed up during the design of a trustworthy block cipher so that cipher text invaders cannot determine the bits' initial sequence. This confusion is made possible by a strong S-box construction that establishes a nonlinear linkage between plaintext bits and cipher text bits. The linear probability described in Eq. (4) is used to assess this algebraic feature of an S-box^40^.

4 $$ LP = max_{{t_{x,} t_{y} \ne 0}} \left| {\frac{{\# \left\{ {x \in X|x.t_{x} = f\left( x \right).t_{y} } \right\}}}{{2^{n} }} - \frac{1}{2}} \right| $$

Here, $t_{x}$ denotes input mask and $t_{y}$ denotes output mask. If the relationship among the input and output bits is linear, the LP value for the S-box will be greater and attackers can easily perform linear cryptanalysis. The predicted S-box has an LP score of 0.125, which is a low enough number to withstand linear cryptanalysis. Therefore, the suggested S-box has a good chance of defeating such cryptanalytic attempts. Table 8 compares the LP values of a few existing S-boxes and predicted S-box. Comparing the proposed S-box to other existing S-boxes, it is clear that it has excellent durability.

### Differential uniformity (DU)

**Figure 3:**
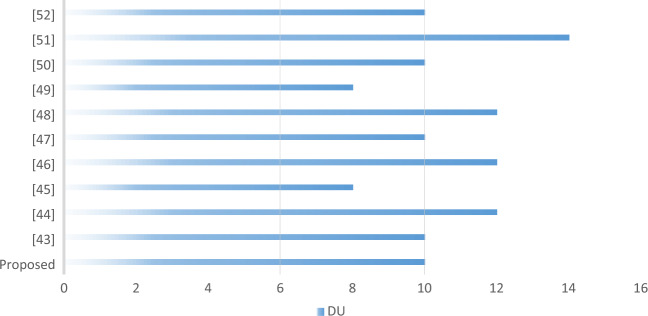
A graphical comparison between DU values of various S-boxes.

The attackers can determine the full or incomplete plaintext or secret key by differential attacks^41^. Analysts assess the differential uniformity (DU) of S-boxes to determine this disparity. An S-box's DU should be relatively small to withstand differential cryptanalysis. Table 9 provides the DU values of the constructed S-box, which were determined using Eq. (3). The highest possible DU score for the predicted S-box is 10. As a result, the differential probability (DP) value equates to 0.039, indicating that the predicted S-box provides respectable resistance to differential cryptanalysis. The contrast of DP values between the predicted S-box and numerous other S-boxes is shown in Table 8. Figure 3 shows a graphical comparison of our S-box's maximum DU results with those of newly developed S-box construction methods.

**Table 9:** Input/output XOR distribution table of created S-box.

6	8	6	8	6	6	6	6	6	6	6	6	6	6	4	8
6	6	6	6	8	10	6	10	6	8	4	6	6	10	6	6
6	4	6	6	6	6	8	6	8	8	6	6	6	6	8	8
6	8	8	6	6	6	6	4	8	6	8	6	8	6	8	6
8	6	10	6	6	6	6	8	6	8	6	6	6	6	8	8
8	8	8	6	6	10	6	6	8	8	6	6	10	10	6	8
8	6	6	6	6	8	6	8	6	6	6	6	6	8	8	6
6	6	6	6	6	6	6	6	6	6	8	8	6	8	8	6
6	6	6	8	6	6	6	6	6	6	6	6	10	6	6	6
6	6	6	6	6	6	8	8	6	8	6	6	6	10	8	8
6	6	6	8	6	6	6	6	8	10	6	6	6	8	4	6
8	6	6	6	6	6	6	10	8	8	8	10	6	6	8	6
6	6	6	6	6	6	6	6	6	6	6	6	8	8	6	6
8	8	6	8	8	6	6	6	6	6	8	6	6	6	8	6
6	8	6	6	8	8	8	8	6	8	8	6	8	6	8	6
6	8	6	6	8	6	6	6	6	6	10	8	6	6	8	0

## Image encryption

S-boxes are an essential component of image encryption algorithms because they provide non-linearity, confusion, and protection against various cryptographic attacks. They help ensure the confidentiality and security of encrypted images by making it extremely difficult for unauthorized parties to reverse-engineer the encryption process or recover the original image without the correct decryption key. Image encryption analysis involves evaluating the security and effectiveness of an image encryption scheme to ensure that it meets the desired security goals. This section carefully analyzes the robustness of the created S-box given in Table 4 in image encryption using several standards for judging the cryptographic potency of substitution boxes.

### Majority Logic Criterion (MLC)

Using various images, the Majority logic criterion performs several statistical analyses to determine the statistical robustness of the S-box in image encryption^58^. The distortion that the encryption process generates in the picture determines how effective the suggested method is. To examine the statistical characteristics, different analyses are required. Correlation, entropy, contrast, homogeneity, and energy are a few of these analyses. JPG images of Cameraman, Pepper, and Baboon taken form the article^65^ are used for MLC analysis. The outcome of image encryption using the suggested S-box and histograms is shown in Table 10. Going forward, Tables 11, 12 and 13 contrast the outcomes of this analysis with those of other well-known S-boxes. These findings suggest that the suggested S-box is sufficient to be included in the algorithms created for the safe transfer of information and data and is appropriate for encryption applications.

**Table 10:**
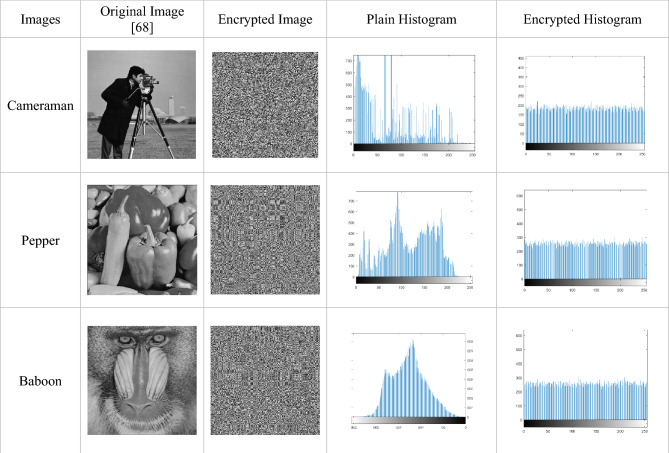
Original and encrypted images with their histograms.

**Table 11:** MLC comparison of Cameraman’s image using the proposed S-box with various other S-boxes.

S-boxes	Contrast	Energy	Homogeneity	Entropy
^59^	10.5367	0.0156	0.3885	7.9956
^14^	10.4236	0.0156	0.3906	7.9956
^60^	10.4901	0.0156	0.3896	7.9957
^61^	10.4966	0.0156	0.3907	7.9959
^62^	10.4366	0.0157	0.3895	7.9963
^15^	10.5685	0.0156	0.3860	7.9972
^63^	10.4659	0.0156	0.3889	7.9958
Proposed	10.4166	0.0156	0.3896	7.9969

**Table 12:** MLC comparison of Baboon image using proposed S-box with various other S-boxes.

S-boxes	Contrast	Energy	Homogeneity	Entropy
^59^	10.4087	0.0157	0.3813	7.9970
^14^	10.3977	0.0157	0.3839	7.9974
^60^	10.5391	0.0157	0.3833	7.9971
^61^	10.4441	0.0157	0.3994	7.9973
^62^	10.6010	0.0157	0.3958	7.9967
^15^	10.5229	0.0157	0.3849	7.9974
^63^	10.3853	0.0157	0.3994	7.9971
Proposed	10.4775	0.01571	0.3810	7.9972

**Table 13:** MLC comparison of Pepper image using proposed S-box with various other S-boxes.

S-boxes	Contrast	Energy	Homogeneity	Entropy
^59^	10.7951	0.0157	0.3809	7.9971
^14^	10.7558	0.0157	0.3818	7.9967
^60^	10.7624	0.0157	0.3812	7.9975
^61^	10.4441	0.0157	0.3994	7.9973
^62^	10.9455	0.0157	0.3822	7.9975
^15^	10.0903	0.0157	0.3994	7.9974
^63^	10.9144	0.0157	0.3823	7.9972
Proposed	10.7321	0.0157	0.3812	7.9975

### Peak Signal-to-Noise Ratio (PSNR)

The Peak Signal-to-Noise Ratio is a metric used to quantify the quality of a reconstructed or compressed signal, typically an image or video, compared to the original, uncompressed signal. PSNR is commonly used in various fields such as image processing, video compression, and signal processing to assess the fidelity of the reconstructed data. The PSNR value is calculated as the ratio of the peak signal power to the noise power, typically expressed in decibels (dB). A higher PSNR value indicates better quality, as it means that the reconstructed signal is closer to the original signal and has less distortion or noise.

The formula for calculating PSNR is:

$$ PSNR = 10 \cdot log_{10} \left( {\frac{{R^{2} }}{MSE}} \right) $$

where R is the maximum possible pixel value of the image and MSE is the Mean Squared Error. The PSNR results of encrypted images are given in Table 14.

**Table 14:** PSNR, MSE, NPCR, UACI results of encrypted images.

Images	PSNR ( dB)	MSE	NPCR (%)	UACI (%)
Cameraman	7.33	0.1328	99.6347	35.1789
Baboon	9.72	0.1067	99.5758	27.3309
Pepper	8.87	0.1298	99.5865	29.4609

### Mean Squared Error (MSE)

Mean Squared Error is a common metric used to measure the average squared difference between the values predicted by a model or estimation technique and the actual values. The MSE test is a mathematical calculation used for evaluating the performance of models or estimators, especially in the context of regression analysis or image processing. In the context of image processing and compression, MSE is often used to quantify the quality of a reconstructed or compressed image compared to the original image. It provides a numerical value that represents the average squared difference in pixel values between the two images. A lower MSE indicates better image fidelity, as it implies that the reconstructed image is closer to the original.

The formula for calculating MSE is as follows:

$$ MSE = \frac{1}{N}\mathop \sum \limits_{i = 1}^{N} \left( {X_{i} - Y_{i} } \right)^{2} $$

where N is the total number of pixels, $X_{i}$ represents the pixel value in the original and $Y_{i}$ represents the corresponding pixel value in the reconstructed or compressed image. The MSE results of encrypted images are given in Table 14.

### Normalized Pixel Change Rate (NPCR)

Normalized Pixel Change Rate is a metric used to evaluate the quality and security of image encryption or data hiding techniques. It is commonly used in the field of information security and cryptography, particularly when assessing the performance of encryption algorithms applied to images. The result is usually expressed as a percentage. In cryptographic applications, a high NPCR value (close to 100%) is desired because it indicates that a small change in the plaintext image results in a significant change in the encrypted image, making it harder for an attacker to deduce information about the original image from the encrypted version.

The formula for calculating NPCR is as follows:

$$ NPCR = \frac{{N_{c} }}{N} \times 100\% $$

where $N_{c}$ is the total number of pixels for encrypted images and $N$ is the total number of pixels in the plain image. The NPCR results of encrypted images are given in Table 14.

### Unified Average Changing Intensity (UACI)

Unified Average Changing Intensity is a metric used to evaluate the quality and security of image encryption or data hiding techniques. The UACI metric measures the average change in pixel intensity values for encrypted images, typically generated by applying an encryption algorithm. A lower UACI value is generally considered better, indicating that the encryption algorithm produces smaller changes in pixel values between the two images.

The formula for calculating UACI is as follows:

$$ UACI = \frac{1}{N}\mathop \sum \limits_{i = 1}^{N} \frac{{\left| {X_{i} - Y_{i} } \right|}}{R} \times 100\% $$

where $N$ is the total number of pixels in the images, $X_{i}$ represents the pixel value in the original image, $Y_{i}$ represents the corresponding pixel value in the encrypted image and *R* is the maximum possible pixel. The UACI results of encrypted images are given in Table 14.

### Complexity analysis

This section investigates the computational complexity of the suggested encryption approach, which involves partitioning the image into Most Significant Bits (MSBs) and Least Significant Bits (LSBs). Each pixel in the image is divided into two sub-blocks with a constant time complexity of $O(1)$. The initial stage necessitates $O(M\times N)$ bit operations where $(M\times N)$ is the dimension of the plain image. The substitution module operates in linear time as well. Because all of the algorithm's modules run in linear time, the scheme's overall computational complexity is $O\left( {M \times N} \right)$. In comparison to previous algorithms^64^, the computational time complexity for creating the cross-coupled chaotic sequence for one round operation is $O\left( {2 \times M_{x} } \right)$, where $M_{x}$ is the maximum value of $M_{1}$ and $M_{2}$. The row-column permutation stage has a temporal complexity of $O\left( {M_{1} \times N_{1} } \right)$, while the row-column diffusion operation has a computational complexity of $8\left( {M_{1} + N_{1} } \right)$. As a result, the overall total computational time complexity of the encryption technique^64^ is $O\left( {2M_{x} + 9(M_{1} + N_{1} } \right)$, which is nearly equivalent to the suggested algorithm.

### Correlation

Correlation analysis in image encryption often involves examining the correlation between different pixel pairs in both the original and encrypted images. This can include analyzing vertical, horizontal, and diagonal correlations. Vertical Correlation calculates the vertical correlation by comparing pixel values vertically in the original and encrypted images, Horizontal Correlation calculates the horizontal correlation by comparing pixel values horizontally in the original and encrypted images, and Diagonal Correlation calculates the diagonal correlation by comparing pixel values diagonally in the original and encrypted images. Each of these correlations provides insights into how well the encryption algorithm disrupts spatial relationships in the image. These correlation results of encrypted images are given in Table 15.

**Table 15:** Correlation results of encrypted images.

Images	Vertical correlation	Horizontal correlation	Diagonal correlation
Cameraman	0.0060	0.0048	− 0.0014
Baboon	− 0.0002	− 0.0012	0.0010
Pepper	− 0.0012	0.0000	0.0006

## Conclusion

In this work, we presented a novel and simple text-theoretical method for the creation of S-boxes. Also, we constructed a sample S-box using the word “UNITY” as an example. When compared to current S-boxes, this newly designed S-box’s algebraic features are strong enough to resist linear and differential attacks, and its performance is superior to that of many other S-boxes. Especially, the mean NL score of the proposed S-box is 111.5, which is extremely high compared to the other S-boxes. The specimen S-box is also utilized in image encryption for images of Cameraman, Pepper, and Baboon. To evaluate the performance of the specimen S-box in image encryption, various statistical analyses such as PSNR, MSE, NPCR, UACI, and MLC are used. In particular, low correlation values of -0.0012, 0.0000, and 0.0006 for vertical, horizontal, and diagonal correlation, respectively, of Pepper image demonstrate the robustness of encryption utilizing the specimen S-box. In future development, this proposed method for S-box construction can be used for dynamic S-box construction using any sentence or paragraph.

## Data Availability

The datasets used during the current study available from the corresponding author on reasonable request.
